# Do early neural correlates of visual consciousness show the oblique effect? A binocular rivalry and event-related potential study

**DOI:** 10.1371/journal.pone.0188979

**Published:** 2017-12-12

**Authors:** Bradley N. Jack, Urte Roeber, Robert P. O’Shea

**Affiliations:** 1 Discipline of Psychology, School of Health and Human Sciences, Southern Cross University, Coffs Harbour, Australia; 2 School of Psychology, UNSW Sydney, Sydney, Australia; 3 Institute for Psychology, University of Leipzig, Leipzig, Germany; 4 Discipline of Biomedical Science, University of Sydney, Sydney, Australia; 5 School of Psychology and Exercise Science, Murdoch University, Perth, Australia; Monash University, AUSTRALIA

## Abstract

When dissimilar images are presented one to each eye, we do not see both images; rather, we see one at a time, alternating unpredictably. This is called binocular rivalry, and it has recently been used to study brain processes that correlate with visual consciousness, because perception changes without any change in the sensory input. Such studies have used various types of images, but the most popular have been gratings: sets of bright and dark lines of orthogonal orientations presented one to each eye. We studied whether using cardinal rival gratings (vertical, 0°, and horizontal, 90°) versus oblique rival gratings (left-oblique, –45°, and right-oblique, 45°) influences early neural correlates of visual consciousness, because of the oblique effect: the tendency for visual performance to be greater for cardinal gratings than for oblique gratings. Participants viewed rival gratings and pressed keys indicating which of the two gratings they perceived, was *dominant*. Next, we changed one of the gratings to match the grating shown to the other eye, yielding binocular fusion. Participants perceived the rivalry-to-fusion change to the dominant grating and not to the other, *suppressed* grating. Using event-related potentials (ERPs), we found neural correlates of visual consciousness at the P1 for both sets of gratings, as well as at the P1-N1 for oblique gratings, and we found a neural correlate of the oblique effect at the N1, but only for perceived changes. These results show that the P1 is the earliest neural activity associated with visual consciousness and that visual consciousness might be necessary to elicit the oblique effect.

## Introduction

One part of the neuroscience of consciousness is the quest to identify neural processes that correlate with phenomenal consciousness [1]. We set out to study those processes using binocular rivalry and the oblique effect.

### Binocular rivalry

What happens when the two eyes view dissimilar images? A remarkable phenomenon, binocular rivalry, ensues in which one of the images is dominant—it reaches visual consciousness and is perceived—whereas the other is suppressed—it does not reach visual consciousness and is not perceived—and visual consciousness of one or the other image alternates unpredictably between the two images [2–7]. For example, when one eye views a horizontal grating—a set of bright and dark horizontal lines—and the other eye views a vertical grating—a set of bright and dark vertical lines—an observer usually perceives one grating, say, horizontal, for a few seconds, then the other, vertical grating for a few seconds, then the horizontal grating again, and so on, for as long as one cares to look. Binocular rivalry is a powerful tool for studying neural activity associated with visual consciousness, because perception of one or the other image changes without any change in the physical properties of those images [1,8,9].

Binocular rivalry can occur between any two images, provided that they are sufficiently dissimilar such that they cannot be combined via binocular fusion—the process yielding singleness of vision of some combination of the images in the two eyes [7,10]. For example, binocular rivalry can be experienced with simple stimuli, such as gratings (contour rivalry; [5]) or colours (colour rivalry; [11]), or with more complex stimuli, such as pictures of faces and houses (complex rivalry; [12]). Gratings are the most popular type of stimuli used to instigate binocular rivalry, possibly because they stimulate simple cells in V1 that are tuned for orientation [13–15], allowing one to study low-level processing [16] free from higher-level influences [17], such as semantics and emotion.

Binocular rivalry is typically explained via neural adaptation and reciprocal inhibition [18]. According to this explanation, the neurons processing each image are involved in reciprocal inhibition such that those tuned for the dominant image are active while those tuned for the suppressed image are inhibited. Because the neurons processing the dominant image are adapting whereas the neurons processing the suppressed image are recovering from adaptation, eventually a tipping point is reached whereby the activity of the two sets of neurons is about equal, requiring only a small nudge, such as from internal noise or from an eye movement, to reverse the balance of inhibition. This leads to an abrupt increase in the activity of the neurons processing the suppressed image as well as a sudden decrease in the activity of the neurons processing the dominant image, forcing a change in visual consciousness. The general principles of this explanation exist in most models of binocular rivalry [19–36].

### Early neural correlates of visual consciousness

Our interest in binocular rivalry is to use it to study early neural correlates of visual consciousness. By this, we mean neural processes that occur in the first 250 ms after the onset of a visual stimulus that differ between conditions in which observers can perceive a visual stimulus versus conditions in which observers cannot perceive a visual stimulus, even though it is projected onto the retina [1,8,9]. To accomplish this, we used the excellent temporal resolution of the electroencephalogram (EEG) and event-related potentials (ERPs), which are in the order of milliseconds, we identified the P1 and N1 components of ERPs, both of which are thought to index early sensory and perceptual processes [37–39], and we used an ERP paradigm pioneered by Kaernbach et al. [40].

Kaernbach et al. [40] presented a left-oblique (–45° from vertical) grating to one eye and a right-oblique (45° from vertical) grating to the other eye, yielding binocular rivalry. They asked their participants to press keys indicating which of the two gratings was dominant. After doing so for at least 10–15 seconds, Kaernbach et al. [40] changed one of the gratings to match the grating shown to the other eye, yielding binocular fusion. We call this event a *rivalry-to-fusion change*. Because of binocular rivalry, if the change was to the dominant grating, then the rivalry-to-fusion change was perceived—we call this a *perceived change*—whereas if the change was to the suppressed grating, then the rivalry-to-fusion change was not-perceived—we call this a *not-perceived change*.

Kaernbach et al. [40] found two neural correlates of visual consciousness: a bigger N1 and a bigger P3 from a perceived change than from an identical not-perceived change. The P3 occurs between 300 and 600 ms and is thought to index task-relevant change-detection and response preparation and execution [41], so it is not a surprising finding, because participants’ task was to release the key when they saw a change. The N1, however, qualifies as an early neural process, because it occurs between 140 and 200 ms and is thought to index sensory, perceptual, and attentional processes [37–39]. The N1 and P3 correlates of visual consciousness have been found with steady rival gratings [42], flickering rival gratings [42], and coloured rival gratings [43].

Roeber and Schröger [42] extended the basic finding to include an earlier neural correlate of visual consciousness: a bigger P1 from a perceived change than from a not-perceived change. The P1 is an early neural correlate of visual consciousness, because it occurs at about 100 ms and is thought to index sensory and perceptual processes [37–39]. The basic P1 finding has since been found by Veser et al. [43] and Roeber et al., [44,45], as well as in the reanalysed data of Kaernbach et al. [40] by Veser et al. [43]. Furthermore, Roeber et al. [45] localised their P1 correlate of visual consciousness to the ventrolateral occipito-temporal cortex, an area of the brain thought to amplify the processing of visual information for visual consciousness [46,47].

### Oblique effect

The oblique effect refers to the relative decrease in perceptual performance for oblique stimuli (i.e., stimuli having orientations that are diagonal; e.g., left-oblique, –45°, and right-oblique, 45°) than for cardinal stimuli (i.e., stimuli having orientations that are vertical, 0°, or horizontal, 90°; [48]). The classical finding is that participants perform better on spatial acuity tasks when the stimuli are aligned to cardinal orientations than to oblique orientations [49–54]. As summarised by Appelle [48] and Li et al., [55], the oblique effect has been found in humans and animals, including the cat, monkey, rabbit, pigeon, goldfish, rat, squirrel, and octopus, for a range of perceptual tasks, including contrast sensitivity, orientation discrimination, and vernier acuity.

Converging evidence suggests that the oblique effect has its neural basis in visual cortex [48,55]. For instance, physical properties of the visual system, such as asymmetric optics and sparser photoreceptor packing in the retina along oblique angles, do not significantly contribute to the oblique effect [52,56], suggesting that the oblique effect arises after the retina. Moreover, single-cell recordings of V1 from the cat [15,57–61] and the monkey [62–64] show that fewer neurons are tuned to oblique stimuli than to cardinal stimuli, suggesting that the oblique effect is a function of the number of cells tuned for specific orientations. In humans, the blood oxygen level-dependent (BOLD) signal in V1 is higher [65] and the amplitudes of ERPs over occipital regions of the brain are bigger [66–72] when observers view cardinal stimuli than when they view oblique stimuli. Thus, the oblique effect has its neural basis in visual cortex.

### Present study

In the experiment we report here, we set out to answer two questions:

1. To date, all of the experiments that have used Kaernbach et al.’s [40] paradigm have used left- and right-oblique gratings as stimuli. Do the results of Kaernbach et al. [40] apply to experiments that use cardinal gratings? Indeed, this question highlights a much larger problem in the literature: most psychophysical studies of binocular rivalry have tended to use cardinal gratings [73–85], whereas most electrophysiological studies have tended to use oblique gratings [40,42–45,86–90]. This is problematic, because using only one set of stimuli for one methodology and a different set of stimuli for a different methodology leaves open the question as to whether conclusions can be generalised across methodologies. We sought to address this.
2. Does the oblique effect require visual consciousness? Recently, Takács et al. [91] found that infrequent and unpredictable changes in the orientation of task-irrelevant gratings elicited a bigger visual mismatch negativity (vMMN), an ERP component thought to index pre-attentive visual change detection [92–94], from cardinal gratings than from oblique gratings. They concluded that the oblique effect does not require attention. Although attention and visual consciousness are different processes that perform separate functions in the brain [1,95–99], the results of Takács et al. [91] prompted us to consider the possibility that the oblique effect may not require visual consciousness. To the best of our knowledge, this has never been tested. We sought to address this.

In summary, we have two purposes: First, we set out to determine whether the results of Kaernbach et al. [40] apply to experiments that use cardinal gratings. Second, we set out to determine whether the oblique effect influences early neural correlates of visual consciousness. To accomplish this, we used an ERP paradigm similar to that of Kaernbach et al. [40], and we compared ERPs from cardinal gratings with ERPs from oblique gratings, and ERPs from perceived changes with ERPs from not-perceived changes.

## Materials and methods

### Ethics statement

The study was approved by Southern Cross University’s Human Research Ethics Committee (ECN-11-149) and was conducted in accordance with the ethical standards laid down in the Declaration of Helsinki [100]. All participants gave written informed consent prior to the experiment.

### Participants

Seventeen volunteers participated in our study. There was no reward or financial incentive offered to participate. All participants had normal or corrected-to-normal visual acuity in both eyes and showed normal binocular rivalry in a 12-minute pre-test session. Data of two participants were excluded from further analyses, because fewer than 50 epochs for any ERP remained after data pre-processing. Mean (*SD*) age of the remaining 15 participants, of whom five were male, was 23 (5) years. We conducted a power analysis of the oblique effect prior to collecting any data based on the effect size reported in Takács et al. [91] of η^2^ = .23: to find a power of 0.8 with α = .05, we needed a sample size of 10.

### Apparatus

The experiment was conducted in the EEG Research Laboratory at Southern Cross University, Coffs Harbour, Australia, in a sound-attenuated (42 dB) room with the display of the stimuli providing the only light. During the experiment, each participant sat in a chair at a desk with his or her head stabilized by a chin-and-forehead rest. Stimuli were presented on a Samsung 2233RZ monitor (1024 × 768 pixels at 60 Hz) at a viewing distance of 57 cm when viewed through a mirror stereoscope (Screenscope-SA-200-Monitor-Type). The experiment was controlled by a Macintosh Mini running specially written Matlab scripts using the Psychophysics Toolbox [101–103]. Participants responded using two keys on a response keypad.

The EEG was recorded from 58 Ag/AgCl active electrodes placed according to the extended 10–20 system (AF7, AF3, AF4, AF8, F7, F5, F3, F1, Fz, F2, F4, F6, F8, FT7, FC5, FC3, FC1, FC2, FC4, FC6, FT8, T7, C5, C3, C1, Cz, C2, C4, C6, T8, TP7, CP5, CP3, CP1, CPz, CP2, CP4, CP6, TP8, P7, P5, P3, P1, Pz, P2, P4, P6, P8, PO9, PO7, PO3, POz, PO4, PO8, PO10, O1, Oz, O2) and referenced to FCz, with the ground at AFz. A vertical electrooculogram (EOG) was recorded by placing an electrode above (we used FP2) and below the right eye; a horizontal EOG was recorded by placing an electrode on the outer canthi of each eye. We also placed an electrode on each earlobe. The sampling rate of the EEG was 500 Hz and the online filtering was 1,000 Hz.

### Stimuli

Binocular-rivalry stimuli were annulus-shaped patches of black (0.40 cd/m^2^) and white (86.67 cd/m^2^) sine-wave gratings on a mean-luminance (43.54 cd/m^2^) grey background. The grating presented to one eye could be vertical (0°), in which case the grating presented to the other eye was horizontal (90°), or left-oblique (–45°), in which case the grating presented to the other eye was right-oblique (45°). The gratings had a spatial frequency of 1.6 cycles per degree, a mean luminance of 43.54 cd/m^2^, and a Michelson contrast of .99. The outer diameter of the gratings was 3.2° of visual angle; the inner diameter was 0.67°. The central area contained a central red fixation cross of 0.3° with a line width of 0.1°. The gratings were surrounded by three white fusion rings; these served to lock vergence. The outer diameter of the largest ring was 6.4°. Each ring had a line width of 0.05° and was 0.3° from its neighbour.

### Design and procedure

The experiment consisted of 20 blocks of about 4 minutes each. In half of them, participants were presented with cardinal stimuli—a horizontal grating to one eye and a vertical grating to the other eye; in the other half, participants were presented with oblique stimuli—a left-oblique grating to one eye and a right-oblique grating to the other eye. The order of the blocks was approximately counterbalanced over participants. Each block contained 24 trials comprising six repetitions of the factorial combination of two eye-orientation arrangements (e.g., left-eye vertical and right-eye horizontal versus left-eye horizontal and right-eye vertical) and two eye-change arrangements (e.g., left-eye grating changed versus right-eye grating changed). The order of trials was random and different within each block, as well as different for each participant.

**Fig 1:**
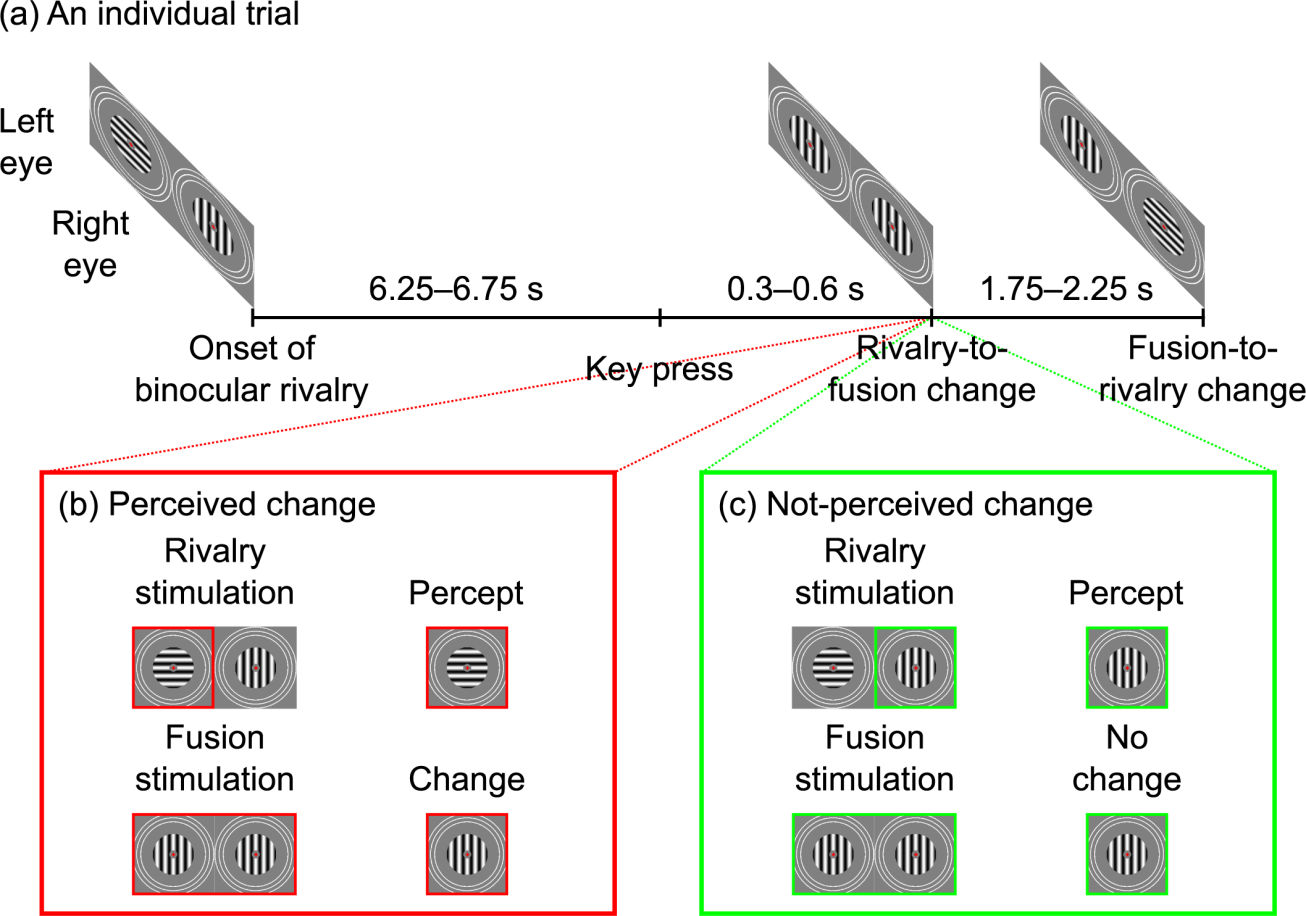
Schematic illustration of one trial of our experimental procedure, based on that described in Kaernbach et al. [40]. (a) A trial comprised a display of binocular-rivalry gratings—in this case, a horizontal grating to the left-eye and a vertical grating to the right-eye—for at least 6.25–6.75 s and until the participant’s next key press. The display then continued for a further 300–600 ms before one of the gratings changed to match the grating shown to the other eye, yielding binocular fusion—in this case, the left-eye grating changed from horizontal to vertical. We call this event a rivalry-to-fusion change. This display lasted for 1.75–2.25 s, at which point one of the gratings changed, yielding binocular rivalry—in this case, the right-eye grating changed from vertical to horizontal. We call this event a fusion-to-rivalry change. This event signalled the end of one trial and the beginning of the next. We also ran counterbalancing trials in which the orientations of the gratings were shown to the opposite eye than that illustrated and the rivalry-to-fusion and fusion-to-rivalry changes were to the opposite eye than that illustrated, as well as trials in which the orientations of the gratings were oblique rather than cardinal. (b) Because of binocular rivalry, if the grating that changed was dominant—in this case, the horizontal grating—the observer perceived the change—we call this a perceived change, (c) whereas if the grating that changed was suppressed—in this case, the vertical grating—the observer did not perceive the change—we call this a not-perceived change.

A trial comprised a display of binocular-rivalry gratings for at least 6.25–6.75 s and until the participant’s next key press. The display then continued for a further 300–600 ms before one of the gratings changed to match the grating shown to the other eye, yielding binocular fusion. We call this event a rivalry-to-fusion change. Because of binocular rivalry, if the change was to the dominant grating, then the rivalry-to-fusion change was perceived—we call this a perceived change—whereas as if the change was to the suppressed grating, then the rivalry-to-fusion change was not-perceived—we call this a not-perceived change. The binocular fusion display lasted for 1.75–2.25 s, at which point one of the gratings changed, yielding binocular rivalry. This event signalled the end of one trial and the beginning of the next (see Fig 1).

The participant’s task was to look at the fixation cross in the centre of the grating stimuli, to report binocular rivalry dominance of one or the other grating by pressing down one or another key on the response keypad, to release that key as soon as dominance changed, and to refrain from pressing either key if any combination of the two gratings was perceived. This yielded two events: key presses—when a key was pressed—and key releases—when a key stopped being pressed. We used key presses and releases to determine participants’ mean binocular rivalry dominance duration and to classify rivalry-to-fusion changes as perceived or not-perceived.

### ERP analysis

For data analyses, we re-referenced the EEG data offline to the average of the earlobes, and we filtered the data using a half-amplitude 0.1 to 35 Hz phase-shift free Butterworth filter (48 dB/Oct slope). We extracted the epochs from -100 to 600 ms, and we baseline corrected all epochs to their mean voltage from -100 to 0 ms. We excluded all epochs with signals exceeding peak-to-peak amplitudes of 200 μV at any EEG channel, or of 60 μV at any EOG channel. We computed ERPs separately for each axis (cardinal, oblique) and for each rivalry-to-fusion change (perceived change, not-perceived change) for each participant, and excluded any data sets containing fewer than 50 epochs for any ERP.

We used participants’ key presses and releases to classify rivalry-to-fusion changes as perceived or not-perceived: if the change was to the dominant grating, we classified it as a perceived change; if the change was to the suppressed grating, we classified it as a not-perceived change. Because binocular rivalry alternations are about 450 ms ahead of a key press or release [3], we classified rivalry-to-fusion changes as perceived or not-perceived only if the key continued to be pressed until at least 150 ms after the rivalry-to-fusion change. We chose this time to be consistent with previous studies using this paradigm [40,42–45].

We defined a spatio-temporal region-of-interest (ROI) according to the literature: Roeber and Schröger [42], Veser et al. [43], and Roeber et al. [44,45] reported the P1 correlate of visual consciousness at right occipital electrodes (PO4, PO8, O2) at about 100 ms, and Kaernbach et al. [40] and Roeber and Schröger [42] reported the N1 correlate of visual consciousness at right occipital electrodes between 150 and 250 ms. From here, we inspected the grand-averaged ERPs at right occipital electrodes, and we identified the P1 between 94 and 114 ms and the N1 between 160 and 190 ms. We analysed the mean amplitudes of the ERPs for the spatio-temporal ROIs using repeated-measures ANOVA with factors axis (cardinal, oblique) and percept (perceived change, not-perceived change). We also calculated voltage maps for the temporal ROIs. Finally, we conducted a series of point-by-point *t*-tests to search for effects outside of the temporal ROIs. To correct for multiple comparisons, we only analysed times between 0 and 250 ms, and we required that at least five consecutive data points have an alpha level equal to or less than .025 in order to be considered significant, as suggested by Guthrie and Buchwald [104]. All *t*-tests were two-tailed.

## Results

### Behavioural results

The time between a key press and its subsequent release yielded the time of one episode of binocular rivalry dominance. As expected, the distribution of these times had the typical gamma-like shape [82,105,106]. Mean (*SD*) binocular rivalry dominance duration was 2.02 (0.67) seconds for cardinal gratings and 1.95 (0.65) seconds for oblique gratings; according to a one-way ANOVA on log-transformed data, these times were not significantly different, *F*(1, 14) = 2.12, *p* = .168, η_p_^2^ = .13 (see S1 Dataset–Worksheet 1). This result suggests that our rival gratings produced the hallmarks of binocular rivalry—exclusivity, inevitability, and randomness [2,4,6]—and that our participants’ experiences of binocular rivalry did not differ between cardinal and oblique gratings, meaning that we did not find any evidence for the oblique effect (for similar findings, see [107–113]).

### ERP results

**Fig 2:**
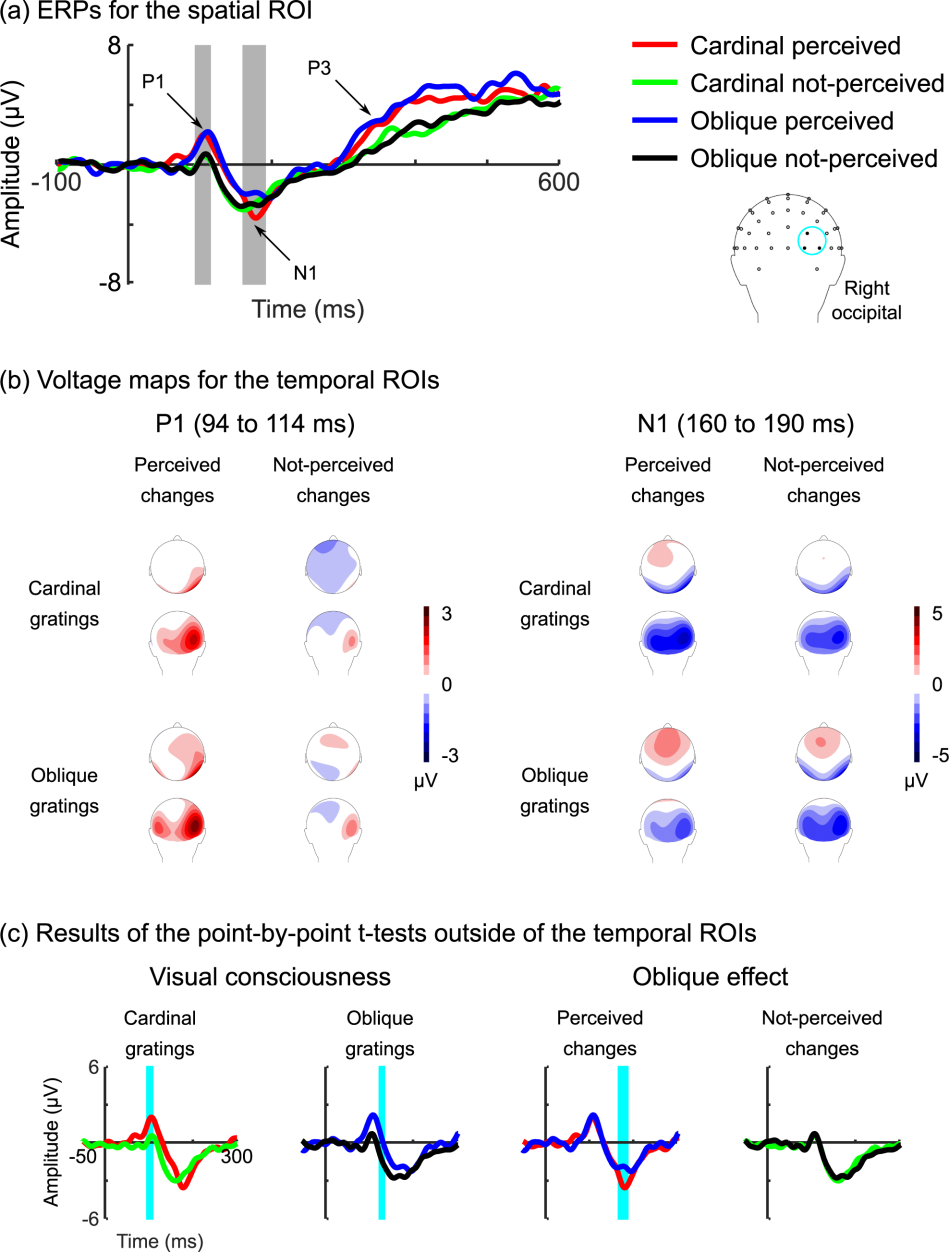
ERPs and voltage maps. (a) ERPs for the spatial ROI. The graph shows time (ms) on the *x*-axis, with 0 indicating the onset of the rivalry-to-fusion change, and voltage (μV) on the *y*-axis, with positive voltages plotted upward. The waveforms show the P1 at about 100 ms, the N1 at about 170 ms, and the P3 from about 350 ms onwards. The grey bars show the P1 and N1 time windows (see text). (b) Voltage maps for the temporal ROIs. (c) Results of the point-by-point *t*-tests outside of the temporal ROIs. The traces are identical to those shown in (a), except that the cyan bars show the time points for which the ERP traces differed significantly (see text).

Fig 2A shows the grand-averaged ERPs at the spatial ROI (to see ERPs outside of the spatial ROI, see S1 Fig). What we see is the P1 at about 100 ms and the N1 at about 170 ms. We also see the P3 from about 350 ms onwards. Note that we do not analyse the P3, because it does not index early sensory and perceptual processing [37–39,41]. In general, the ERP waveforms are similar to previous studies using this paradigm [40,42–45]. Fig 2B shows that there were positive voltages at right occipital electrodes for the P1, whereas for the N1, there were negative voltages at right occipital electrodes and positive voltages at fronto-central electrodes. We consistently see this right-hemisphere bias in many of our binocular rivalry and ERP studies [43,45,92,110].

#### P1

Repeated-measures ANOVA for the P1 time window found a significant main effect of percept, with perceived changes yielding bigger voltages than not-perceived changes, *F*(1, 14) = 7.54, *p* = .016, η_p_^2^ = .35—this is a neural correlate of visual consciousness—and is consistent with that found by Roeber and Schröger [42], Veser et al. [43], and Roeber et al., [44,45], as well as in the reanalysed data of Kaernbach et al. [40] by Veser et al. [43]. However, there was no difference between cardinal and oblique gratings, *F*(1, 14) = 0.12, *p* = .731, η_p_^2^ = .01, and the interaction between axis and percept was not significant, *F*(1, 14) < 0.01, *p* = .988, η_p_^2^ < .01 (see S1 Dataset and S1 Table). These results suggest that the P1 does not index the oblique effect.

#### N1

Repeated-measures ANOVA for the N1 time window failed to find any significant differences. In particular, there was no difference between cardinal and oblique gratings, *F*(1, 14) = 2.47, *p* = .138, η_p_^2^ = .15, or between perceived and not-perceived changes, *F*(1, 14) = 0.02, *p* = .888, η_p_^2^ < .01. Furthermore, the interaction between axis and percept was not significant, *F*(1, 14) = 2.49, *p* = .137, η_p_^2^ = .15 (see S1 Dataset and S1 Table). These results suggest that the N1 does not index visual consciousness (for similar findings, see [43–45]) or the oblique effect.

#### Point-by-point *t*-tests

Fig 2C shows the results of the point-by-point *t*-tests. We found that there were differences between perceived and not-perceived changes for cardinal gratings from 96 to 108 ms, *t*(14) = 2.76, *p* = .015, and for oblique gratings from 122 to 132 ms, *t*(14) = 2.60, *p* = .021—these are neural correlates of visual consciousness at the P1 and P1-N1. We also found that there were differences between cardinal and oblique gratings for perceived changes from 164 to 184 ms, *t*(14) = 3.76, *p* = .002—this is a neural correlate of the oblique effect at the N1. There were no differences between cardinal and oblique gratings for not-perceived changes (see S1 Dataset). These results suggest that the oblique effect does not influence early neural correlates of visual consciousness, but that visual consciousness might be necessary to elicit the oblique effect.

## Discussion

We set out to determine whether the results of Kaernbach et al. [40] apply to experiments that use cardinal gratings, and to determine whether the oblique effect influences early neural correlates of visual consciousness. To accomplish this, we used an ERP paradigm similar to that of Kaernbach et al. [40], and we compared ERPs from cardinal gratings with ERPs from oblique gratings, and ERPs from perceived changes with ERPs from not-perceived changes. We found neural correlates of visual consciousness at the P1 for both sets of gratings, as well as at the P1-N1 for oblique gratings, and we found a neural correlate of the oblique effect at the N1, but only for perceived changes. These results show that the P1 is the earliest neural activity associated with visual consciousness and that visual consciousness might be necessary to elicit the oblique effect.

We found the P1 correlate of visual consciousness for both cardinal and oblique gratings, and we found that the P1 correlate of visual consciousness does not index the oblique effect. This finding confirms and extends upon those of Roeber and Schröger [42], Veser et al. [43], and Roeber et al., [44,45], as well as the reanalysed data of Kaernbach et al. [40] by Veser et al. [43]. Furthermore, the timing of our P1 correlate of visual consciousness agrees with that estimated using other techniques that have manipulated visual consciousness, such as backward masking and bistable images [114–122]. These results provide converging evidence for the conclusion that the P1 is the earliest neural activity associated with visual consciousness.

However, Railo et al. [123] have argued that the P1 correlate of visual consciousness might be confounded with attention, because the P1 is enhanced by both spatial [124] and feature-based attention [125]. They argue that when a rivalry-to-fusion change is made to the dominant grating, it is the attended and perceived grating that changes, whereas when an identical change is made to the suppressed grating, it is the unattended and not-perceived grating that changes. We disagree with Railo et al. [123] for at least three reasons:

1. The suppressed grating is attended during binocular rivalry. Because the gratings were perceptually overlaid, spatial attention did not differ between the perceived and not-perceived conditions, and because participants were instructed to press down one key when one grating was dominant and a different key when a different grating was dominant, feature-based attention also did not differ between the perceived and not-perceived conditions. In this, binocular rivalry dominance is “attention with consciousness” [96], whereas binocular rivalry suppression is “attention without consciousness” [96]. Therefore, visual consciousness, not attention, alternates back and forth during binocular rivalry.
2. Recently, Roeber et al. [44] showed that the amplitude of the P1 elicited by a rivalry-to-fusion change is essentially the same whether or not attention is on the gratings. This means that even if Railo et al. [123] are correct in assuming that attention is present for a perceived change and absent for a not-perceived change, attention’s influence on the P1 during binocular rivalry is not statistically significant.
3. Railo et al. [123] argued that Veser et al.’s [43] failure to find a P1 for not-perceived changes is consistent with their claim that this paradigm confounds attention and visual consciousness. Although they are correct, their argument ignores the reanalysis of the results of Kaernbach et al. [40] by Veser et al. [43], as well as the results of Roeber and Schröger [42] and Roeber et al., [45], all of which show a P1 from not-perceived changes. Since Railo et al. [123] published their review, two more studies using this paradigm have shown a P1 to not-perceived changes: Roeber et al. [44] and the present study.

Railo et al. [123] have also argued that the P1 is too early to be the neural process that gives rise to visual consciousness. Instead, they argued that the P1 reflects preconscious processes, such as sensory processing, and that visual consciousness emerges at the N1, which they call the “visual awareness negativity” (VAN) [123]. However, our results are problematic for Railo et al.’s [123] argument, because we found that the N1 correlate of visual consciousness may not be particularly robust. Specifically, we failed to find the N1 correlate of visual consciousness for both sets of gratings. It is also worth mentioning that Veser et al. [43] and Roeber et al., [44,45] failed to find the N1 correlate of visual consciousness to orientation changes (they did not analyse the N1, but visual inspection of their figures suggests that a difference between perceived and not-perceived changes is unlikely). Perhaps a different way to think about all of this is that either the P1 or the N1/VAN can precede the emergence of visual consciousness, and that one or the other (or both [40,42]) is a necessary condition for such visual consciousness.

We found a neural correlate of the oblique effect at the N1, around 170 ms, but only when the rivalry-to-fusion change was perceived. This is noteworthy for at least two reasons:

1. Our results suggest that visual consciousness might be necessary to elicit the oblique effect. This is a curious finding, because it goes against the notion that the oblique effect is a basic phenomenon of the visual system [48,55], independent of psychological constructs such as visual consciousness. However, because we have provided rather limited evidence for this conclusion, before we believe it, we would like to see further evidence for it. Specifically, we would like to see a replication of our results, and we would like to see this research extended, such as by using other techniques to manipulate visual consciousness. We look forward to reading about this research in the future.
2. Takács et al. [91] found that the oblique effect (also around 170 ms) does not require attention. This might seem to contradict our conclusion that the oblique effect might require visual consciousness. But attention and visual consciousness are different processes that perform separate functions in the brain [1,95–99], and it is possible that these different processes and functions have opposing effects on the neural correlates of the oblique effect. Of course, the only way to show this beyond reasonable doubt is to conduct another experiment that doubly dissociates attention and visual consciousness, similar to those conducted by van Boxtel et al. [98] and Watanabe et al. [99]. We look forward to conducting this experiment in the future.

Consistent with previous research [107–113], we found that our participants’ experiences of binocular rivalry did not differ between cardinal and oblique gratings. That is, we found that our rival gratings produced the hallmarks of binocular rivalry—exclusivity, inevitability, and randomness [2,4,6]. This is an important result because it means that findings and conclusions of psychophysical studies of binocular rivalry, which have tended to use cardinal gratings [73–85], can be generalised to electrophysiological studies, which have tended to use oblique gratings [40,42–45,86–90]. Furthermore, because our ERP results show that the oblique effect is rather subtle, we are confident that findings and conclusions of electrophysiological studies of binocular rivalry can be generalised to psychophysical studies.

However, this prompts the following questions: why did we find weak (as opposed to strong) evidence for the oblique effect? One possible explanation is that the oblique effect can only occur after the inputs from each eye are combined—binocular fusion—and that the oblique effect cannot occur when the inputs do not combine—binocular rivalry [126]. Nevertheless, in the present study, we recorded ERPs from the onset of binocular fusion. So, why did we *still* find weak evidence? Some studies [12,127,128] have shown that the effects of binocular rivalry can continue to be observed in V1, the neural site of both binocular fusion [129] and the oblique effect [55], for a short time after the offset of binocular rivalry stimuli. Indeed, this assumption lies at the heart of all the studies that use this paradigm [40,42–45]; otherwise, why would the P1 (which is thought to have its neural sources in V1 [130–132]) differ between perceived and not-perceived changes from stimuli yielding binocular fusion? All of this is to say that we suspect that it is difficult to elicit the oblique effect during binocular rivalry, simply because binocular rivalry weakens the oblique effect; however, we concede that this is speculation.

In conclusion, we set out to determine whether the results of Kaernbach et al. [40] apply to experiments that use cardinal gratings, and to determine whether the oblique effect influences early neural correlates of visual consciousness. To accomplish this, we used an ERP paradigm similar to that used by Kaernbach et al. [40], and we compared ERPs from cardinal gratings with ERPs from oblique gratings, and ERPs from perceived changes with ERPs from not-perceived changes. We found neural correlates of visual consciousness at the P1 for both sets of gratings, as well as at the P1-N1 for oblique gratings, and we found a neural correlate of the oblique effect at the N1, but only for perceived changes. We conclude that the results of Kaernbach et al. [40] apply to experiments that use cardinal gratings, that the P1 is the earliest neural activity associated with visual consciousness, and that visual consciousness might be necessary to elicit the oblique effect.

## Supporting information

S1 Dataset Behavioural and ERP data. The dataset contains four worksheets: the first shows the mean binocular rivalry dominance durations, the second and third show the mean amplitudes of the P1 and N1 time windows, respectively, at left and right frontal, central, parietal, and occipital electrodes, and the fourth shows the significant time points for the point-by-point t-tests (see text).
(XLSX)

S1 Fig ERPs outside of the spatial ROI. The graphs show the waveforms at left frontal (AF7, AF3, F3), right frontal (AF4, AF8, F4), left central (C5, C3, C1), right central (C2, C4, C6), left parietal (P5, P3, P1), right parietal (P2, P4, P6), left occipital (PO7, PO3, O1), and right occipital (PO4, PO8, O2) electrodes. The grey bars show the P1 and N1 time windows (see text).
(PDF)

S1 Table Statistical analyses. Results of the statistical analysis of the mean amplitudes of the P1 and N1 time windows using repeated-measures ANOVA with factors region (frontal, central, parietal, occipital), hemisphere (left, right), axis (cardinal, oblique), and percept (perceived change, not-perceived change).
(DOCX)
