# Supplementary figures and images for: Do early neural correlates of visual consciousness show the oblique effect? A binocular rivalry and event-related potential study
Source: PLoS One. 2017 Dec 12;12(12):e0188979. doi: 10.1371/journal.pone.0188979 (PMC5726736; doi:10.1371/journal.pone.0188979)

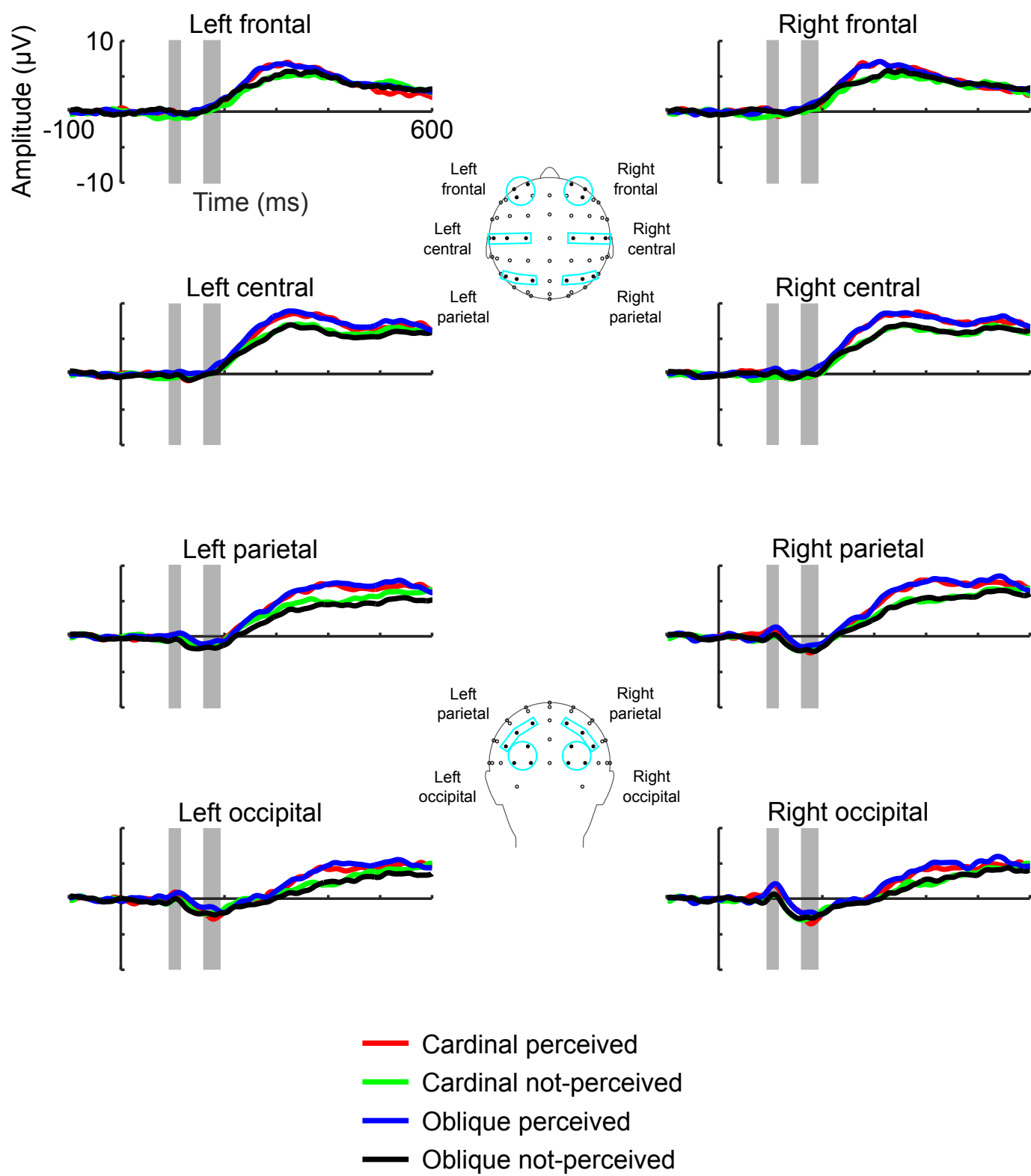

Supplement: S1 Fig — The graphs show the waveforms at left frontal (AF7, AF3, F3), right frontal (AF4, AF8, F4), left central (C5, C3, C1), right central (C2, C4, C6), left parietal (P5, P3, P1), right parietal (P2, P4, P6), left occipital (PO7, PO3, O1), and right occipital (PO4, PO8, O2) electrodes. The grey bars show the P1 and N1 time windows (see text). (PDF) [file pone.0188979.s002.pdf]
