# Supplementary material for: Do early neural correlates of visual consciousness show the oblique effect? A binocular rivalry and event-related potential study
Source: PLoS One. 2017 Dec 12;12(12):e0188979. doi: 10.1371/journal.pone.0188979 (PMC5726736; doi:10.1371/journal.pone.0188979)
Supplement: S1 Table — Results of the statistical analysis of the mean amplitudes of the P1 and N1 time windows using repeated-measures ANOVA with factors region (frontal, central, parietal, occipital), hemisphere (left, right), axis (cardinal, oblique), and percept (perceived change, not-perceived change). (DOCX) [file pone.0188979.s003.docx]

**S1 Table. Statistical analyses.** Results of the statistical analysis of the mean amplitudes of the P1 and N1 time windows using repeated-measures ANOVA with factors region (frontal, central, parietal, occipital), hemisphere (left, right), axis (cardinal, oblique), and percept (perceived change, not-perceived change).

| Factor | *df* | P1 | | |  | N1 | | |
| --- | --- | --- | --- | --- | --- | --- | --- | --- |
|  |  | *F* | *p* | η_p_^2^ |  | *F* | *p* | η_p_^2^ |
| Region (R) | 3, 42 | 2.34 | .087 | .14 |  | 6.97 | **.001** | .33 |
| Hemisphere (H) | 1, 14 | 4.86 | **.045** | .26 |  | 1.82 | .198 | .12 |
| Axis (A) | 1, 14 | 0.54 | .473 | .04 |  | 0.80 | .387 | .05 |
| Percept (P) | 1, 14 | 2.85 | .114 | .17 |  | 1.15 | .301 | .08 |
| R x H | 3, 42 | 2.05 | .122 | .13 |  | 1.57 | .210 | .10 |
| R x A | 3, 42 | 0.92 | .440 | .06 |  | 0.40 | .752 | .03 |
| H x A | 1, 14 | 1.25 | .282 | .08 |  | 1.34 | .267 | .09 |
| R x H x A | 3, 42 | 0.28 | .838 | .02 |  | 0.79 | .508 | .05 |
| R x P | 3, 42 | 6.94 | **.001** | .33 |  | 0.28 | .841 | .02 |
| H x P | 1, 14 | 2.45 | .140 | .15 |  | 0.25 | .622 | .02 |
| R x H x P | 3, 42 | 2.26 | .095 | .14 |  | 0.29 | .834 | .02 |
| A x P | 1, 14 | 0.10 | .762 | .01 |  | 1.30 | .274 | .08 |
| R x A x P | 3, 42 | 0.43 | .735 | .03 |  | 1.72 | .177 | .11 |
| H x A x P | 1, 14 | 0.03 | .860 | < .01 |  | 0.01 | .917 | < .01 |
| R x H x A x P | 3, 42 | 1.54 | .219 | .10 |  | 0.15 | .926 | .01 |

Note: Values highlighted in bold indicate statistical significance (*p* < .05).
